# Supplementary material for: Exploring exercise, emotional eating, and body dissatisfaction in depression risk: Using structural equation modeling
Source: PLoS One. 2025 May 9;20(5):e0322635. doi: 10.1371/journal.pone.0322635 (PMC12063905; doi:10.1371/journal.pone.0322635)
Supplement: Attachment — Submitted filename: Response to Reviewers.docx [file pone.0322635.s003.docx]

Dear Editor and Reviewers,

We are deeply grateful for the time, effort, and invaluable insights you have generously offered in reviewing our manuscript and sincerely look forward to your further review. We are very grateful for your thoughtful and constructive comments, which will make a significant contribution to improving the quality of our work. We have carefully revised the manuscript in response to your suggestions, and the changes have been highlighted in red for your convenience. Below, we provide a detailed response to each comment, outlining the specific revisions we have made.

Thank you very much for giving me the opportunity to revise my manuscript. As requested, I have included the data related to this study, the Study Protocols, and the figures processed by PACE in the supporting information file. Additionally, I have modified the format of my manuscript according to the requirements of PLOS ONE.

**Reviewer** **Comments 1**

**Point 1**

Generally, the manuscript is grammatically well written, but it does need to be proofed for errors and inconsistencies – for example, man instead of male, and a space after the last word and the bracket for citation.

**Response 1**

Thank you very much for pointing out the issues in the details of my manuscript. I have carefully re-examined the full text and made the following revisions: I have replaced all instances of “male and female” with “man and woman.” Additionally, the other two problems you mentioned have also been corrected. In the process, I also identified and fixed some similar issues myself. For example, I had incorrectly written the unit of BMI as “kg/m2,” which I have now correctly revised to “kg/m².”

**Point 2**

The manuscript starts by highlighting the importance of depression among college students, but as you get into the moderators (PA, EES, body dissatisfaction), the justification for the overall study design seems to get a bit lost.

Overall, there’s a thorough discussion, but the relationships between these outcomes gets a little lost. You talk through each relationship individually, but there is not a cohesive explanation of all findings. A careful, thoughtful, well-worded explanation is needed that describes these mediating relationships, how it impacts depression, and then how the reader can use this information. Starting the manuscript, I would assume PA, EES, and body dissatisfaction mediate depression – upon ending the manuscript, it felt like you talked about each individual relationship, but now how they act together on depression.

**Response 2**

Thank you very much for your insightful comments regarding the overall flow and coherence of our manuscript. We appreciate your feedback and agree that a more cohesive explanation of the mediating relationships and their collective impact on depression is needed. This will help readers better understand the overall study design and the practical implications of our findings.

In the final paragraph of the Introduction, we have added the following content: In summary, depression represents a significant mental health issue among college students. Elucidating the factors that contribute to depression is essential for devising effective interventions. Prior research has established physical activity, emotional eating, and body dissatisfaction as key factors influencing mental health. However, the interrelationships among these factors and their collective impact on depression remain to be fully explored. Thus, the present study aims to examine the combined effects of physical activity, emotional eating, and body dissatisfaction on depression risk among Chinese college students using structural equation modeling.

In the final paragraph of the Discussion, we have added the following content: On the whole，exercise significantly mediated the relationship between BMI and depression, accounting for 30% of the total effect. This suggests that physical activity has a substantial impact on reducing depression risk. Evidence indicates that trajectories of lean mass and fat mass remain stable during adolescence [53]. If higher BMI partly reflects higher fat mass, but lean mass represents a body shape preferred by society [24], this implies that exercise may improve overall mental health by enhancing self-esteem. Moreover, exercise also mediated the relationship between body weight dissatisfaction and emotional eating, contributing to 19.8% of the total effect. This indicates that engaging in regular physical activity can help mitigate the negative impact of body dissatisfaction on emotional eating behaviors. Emotional eating behavior partially mediated the relationship between exercise and depression, accounting for 8.5% of the total effect. This may be because 71.3% of the participants had low levels of physical activity, which weakened the association between emotional eating and depression. While this mediating effect is smaller compared to exercise, it still highlights the importance of addressing emotional eating behaviors in interventions aimed at reducing depression risk.

**Point 3**

The abstract is lacking any data, please include specifics that are found in the results section.

**Response 3**

Thank you very much for your comments on the abstract section. I have added data related to the results in the abstract section and revised the relevant sentences as follows: Exercise served as a significant mediator between BMI and depression, as well as between body dissatisfaction and emotional eating, accounting for 30% and 19.8% of the total effects, respectively.

**Point 4**

The first sentence of the abstract is incomplete.

**Response 4**

Thank you very much for your comments on the abstract section. I have put “In order to fill the relevant research gap on the influence of body dissatisfaction on depression in Chinese college students. We used structural equation modeling to explore the relationships between physical activity, body dissatisfaction, dietary behavior and depression.” modified to “In order to fill the relevant research gap on the influence of body dissatisfaction on depression in Chinese college students, we used structural equation modeling to explore the relationships between physical activity, body dissatisfaction, dietary behavior and depression.”

**Point 5**

The sentence starting with “during college years” needs cited

**Response 5**

I have found the sentence you mentioned in the manuscript: During college years, when students do not receive parental supervision, eating behaviors change dramatically, and unhealthy eating behaviors becomes increasingly common, which directly affects students’ emotional state. A relevant reference is added.

**Point 6**

The sentence starting with “in addition to change sin physical activity” – whatever point you’re trying to make her is not fully explained.

**Response 6**

Thank you very much for your careful observation in the introduction section. In order to make the sentences better understood, I reorganized the language. As follows: In addition to alterations in physical activity and eating behaviors, the participants also exhibited romantic needs and expressed a desire to seek a significant other.

**Point 7**

“head portal sensitization” is an unfamiliar recruitment method, please full explain

**Response 7**

Thank you very much for your comments on the careful observation of the method section. I'm sorry that this is caused by my unclear expression. I have revised the manuscript as follows: Recruitment of participants was conducted through oral publicity and distribution of pamphlets in study rooms.

**Point 8**

It says in a few places that participants did 3 questionnaires, but it looks like they did 4 – can you please clarify in the manuscript?

**Response 8**

Thank you very much for your comments on the careful observation of the method section. We apologize for the confusion regarding the measurement of body dissatisfaction. It should be clarified that the SDS scale, C-DEBQ scale, and PRAS-3 scale were the only instruments employed in this study. To prevent body dissatisfaction from being misinterpreted as a scale, I moved this section behind "measure".

We corrected the misunderstanding regarding the calculation of ideal weight. Our ideal weight was self-reported by participants, Ideal BMI was calculated from actual height and self-reported ideal weight, and body dissatisfaction was calculated based on the difference between actual and ideal BMI. We included an article about the method we used to measure body dissatisfaction index, which has been recognized by numerous researchers.

**Point 9**

instead of “man” use “male”

**Response 9**

I have carefully re-examined the full text and made the following revisions: I have replaced all instances of “male and female” with “man and woman.”

**Point 10**

Mouth is the wrong word in the discussion

**Response 10**

Thank you very much for your careful observation in the discussion section. I have changed "mouth" to "month".

**Point 11**

The first paragraph needs to summarize what you found, then the second paragraph can dig into a discussion about one of the primary findings.

**Response 11**

Thank you very much for your comments on the discussion section. I have added the corresponding content in the first paragraph of the discussion. As follows: This study investigates the complex relationships between exercise, body dissatisfaction, emotional eating, and depression among Chinese college students using structural equation modeling. Our results demonstrate that exercise significantly mediates the relationship between BMI and depression, accounting for 30% of the total effect. Additionally, exercise mediates the relationship between body dissatisfaction and emotional eating, contributing to 19.8% of the total effect. Emotional eating behavior also partially mediates the relationship between exercise and depression.

**Reviewer Comments 2**

Major issues with the manuscript are as follows:

**Point 1**

In the methods, the authors note that the model is “satisfactory” and list values related to model fit. The manuscript would be stronger with a table reporting more details about the model fit, as well as text explaining whether the authors considered alternative models and how they settled on this one. An invited review by Kang and Ahn published in Asian Nursing Research in 2021 presents checklists and guiding questions to address during the all stages of SEM-based studies to help improve reporting quality (Kang and Ahn, 2021). They recommend including tables showing goodness of fit indices and estimates of loadings and covariances of measurement errors.

**Response 1**

Thank you very much for your comments on the methods section of the manuscript. Based on your comments, I found the paper you mentioned and reported the relevant fit index. The following additions were added to the manuscript: Moreover, the model fitting index indicated that the structural equation model was satisfactory (AIC = 56057, BIC = 56155, P = 0.013). Table 4 provides examples of reporting results that mainly use fit indices. We used several fit indices to test the fit of the data model: (i) Fit index (CFI, AGFI, GFI, and TLI) >0.95 was considered satisfactory, and values between 0.90 and 0.95 were considered acceptable; (ii) Root mean square Error of Approximation (RMSEA), the range of 0.05-0.08 indicates a sufficient fit; Standardized root mean square residual (SRMR), values < 0.05 indicate a good fit and a range between 0.05 and 0.10 is acceptable.

Table 4. Goodness of Fit Indices

|  | χ² | df | GFI | AGFI | RMSEA | SRMR | CFI | TLI | |
| --- | --- | --- | --- | --- | --- | --- | --- | --- | --- |
| Model | 8.632 | 2 | 0.998 | 0.985 | 0.043 | 0.009 | 0.996 | 0.982 |  |

Note. AGFI = adjusted goodness of fit index; CFI = comparative normed fit index; df = degree of freedom; GFI = goodness-of-fit index; RMSEA = root mean squared error of approximation; SRMR = standardized root mean square residual; TLI = Tucker-Lewis index.

However, in this study, structural equation model only reports the path diagram, which does not involve the measurement variables, so it cannot report estimates of loadings and covariances of measurement errors.

**Point 2**

The authors cite evidence in the introduction and discussion indicating a bi-directional relationship between emotional eating and depression (citations #19, #43). It’s unclear why the model does not include the potential for depression to increase emotional eating since there is reason to believe this relationship exists based on prior literature.

**Response 2**

Thank you very much for your valuable comments. The bidirectional relationship between emotional eating and depression that you mentioned is indeed an important issue. Although our study primarily focused on the impact of emotional eating on depression, we are also aware that depression may, in turn, influence emotional eating behavior. This bidirectional relationship is indeed well-supported by existing literature (as cited in references #19 and #43).

When constructing our model, we mainly considered the following factors:

Our primary objective was to explore the relationships between body dissatisfaction, exercise, emotional eating, and depression, with a particular emphasis on the impact of body dissatisfaction and emotional eating on depression. Therefore, we prioritized the unidirectional relationships among these variables in our model to clearly illustrate the main pathways.

Although we collected extensive data, incorporating bidirectional relationships into the model would have made it overly complex and difficult to interpret. Moreover, given the cross-sectional nature of our data, we were unable to clearly distinguish the direction of causality. Hence, we primarily presented unidirectional relationships in our model.

However, your suggestion is extremely valuable. In future research, we plan to further investigate this bidirectional relationship, especially through longitudinal study designs, which will help us gain a more comprehensive understanding of the dynamic relationship between emotional eating and depression. Additionally, we will consider using more sophisticated statistical methods (such as cross-lagged panel analysis or dynamic systems modeling) in our subsequent studies to better capture this bidirectional relationship.

**Point 3**

In this study the authors use the metric of a body dissatisfaction score, which is calculated as actual BMI minus desired BMI. Observed variables should be measured using tools that have been verified for accuracy and reliability (Kang and Ahn, 2021). It’s unclear if this metric is validated against other accepted measures of body dissatisfaction. The methods section includes a citation for Venckunas, 2016, in the description for collecting information about body dissatisfaction. However, that article does not measure body dissatisfaction or include the body dissatisfaction score metric; it’s a paper about trends in fitness and body size in Lithuanian children. A quick search of the literature for papers using this metric revealed only papers from the author’s lab and one other paper (Meyer, 2024) using that approach. Body dissatisfaction is related to more than someone’s desired weight, which as an indicator itself also doesn’t account for any body image disturbance related to body composition, weight distribution, muscular appearance or “tone,” or other physical attributes. Body dissatisfaction is a cognitive-affective psychological construct (Quittkat, 2019). The actual minus desired BMI metric doesn’t really seem to capture body dissatisfaction in this sense. The metric used by the authors is better defined as a weight discrepancy or BMI discrepancy. Indeed, the other paper that used actual minus desired BMI as an indicator of body dissatisfaction (Meyer, 2024) spent majority of the discussion talking about their findings in the context of discrepancy between current and desired BMI, rather than in the context of the feelings of being dissatisfied with one’s body.

**Response 3**

Thank you for your insightful comments regarding the use of the (Venckunas et al 2016) reference and the measurement of body dissatisfaction. We appreciate your detailed feedback and agree that the reference to (Venckunas et al 2016) was not entirely appropriate in the context of body dissatisfaction measurement. We sincerely apologize for this oversight. To clarify, while (Venckunas et al 2016) focused primarily on trends in physical fitness, their methodology regarding the measurement of BMI and ideal weight provided a valuable methodological reference for our study. We acknowledge that body dissatisfaction is a multidimensional psychological construct that encompasses cognitive, affective, and socio-cultural factors. The BMI discrepancy metric, while not capturing the full complexity of these dimensions, offers significant advantages in terms of simplicity and data availability in large-scale cross-sectional studies. Additionally, the BMI discrepancy can provide a straightforward indication of individuals' cognitive differences regarding their weight.

We will add a description of the measurement method for body dissatisfaction in our study to the limitations section of the manuscript. I have revised the term "body dissatisfaction" to "body weight dissatisfaction" in the manuscript. As follow: The difference between the actual and ideal BMI values was used as the body weight dissatisfaction score, measuring body weight dissatisfaction among Chinese college students.

In the introduction to the manuscript, I added the following: Body dissatisfaction refers to the negative feelings that individuals experience due to inconsistencies between their actual and desired appearance or weight（Blundell, 2024）.Body weight dissatisfaction represents a specific dimension of body dissatisfaction, characterized by a discrepancy between actual and ideal BMI, particularly among individuals with obesity (Meyer, 2024). The following papers were cited separately:

Blundell, E.; De Stavola, B. L.; Kellock, M. D.; Kelly, Y.; Lewis, G.; McMunn, A.; Nicholls, D.; Patalay, P.; Solmi, F. Longitudinal pathways between childhood BMI, body dissatisfaction, and adolescent depression: an observational study using the UK Millenium Cohort Study. *Lancet Psychiatry* **2024**, *11* (1), 47-55. DOI: 10.1016/s2215-0366(23)00365-6

Meyer, E.; Lönnroth, K.; Forsell, Y.; Lagerros, Y. T. Discrepancy between Current, Desired, and Ideal Body Mass Index in Persons with Obesity: A Swedish Population-Based Study. *Obes Facts* **2024**, *17* (1), 72-80. DOI: 10.1159/000535198

**Point 4**

The C-DBEQ and original DBEQ are validated instruments that have been used to quantify emotional eating behaviors in many research studies. However, in 2016, Bongers and Jansen published a review in Frontiers in Psychology that calls into question the validity of the DBEQ and other self-report questionnaires that measure emotional eating as being reflective of increased food intake in response to negative emotions (Bongers, 2016). They argue that evidence does not support a consistent link between emotional eating scores and increased food intake. Additionally, they point out that emotional eating can also occur in response to positive feelings in addition to negative feelings. Instead, they show that high emotional eating scores are related to high scores on several other eating-related scales, and thus may reflect other behaviors or traits, including lack of control, general eating concerns, a tendency to attribute overeating to negative affect, or learned cue reactivity. If interventions towards emotional eating are going to be effective, they must target actual behaviors or traits associated with the alterations in food intake and thus mood and depression risk and so the general concept of emotional eating may not be helpful. There is precedent for using the emotional eating scale used in this study, so a way that the authors could strengthen the manuscript would be to address this in the discussion of their findings. The authors should be cognizant of how well the construct used to measure emotional eating (body dissatisfaction, as well) actually represents the behavior or trait of interest when considering the relationships between emotional eating scores, dissatisfaction with body weight, physical activity levels, and depression risk and incorporate discussion of this potential limitation to the study.

**Response 4**

We sincerely thank the reviewer for raising this critical issue regarding the validity of emotional eating measurement tools. We appreciate the reviewer’s reference to the study by Bongers and Jansen (2016), which indeed questions the relationship between emotional eating scores and actual food intake, highlighting inconsistencies in this association. Their work also emphasizes that emotional eating may not solely correlate with negative emotions but could extend to positive emotions. Furthermore, high emotional eating scores might reflect broader eating-related behaviors or traits, such as loss of control, general dietary concerns, a tendency to attribute overeating to negative emotions, or learned cue reactivity.

In our study, we selected the Chinese version of the DEBQ (C-DEBQ) as the measurement tool for emotional eating due to its established validation in Chinese populations and its widespread use in similar research contexts. While acknowledging the ongoing debate, the C-DEBQ remains one of the most commonly utilized instruments for assessing emotional eating behaviors. Our research primarily focuses on exploring the relationship between emotional eating behaviors and depression risk, rather than evaluating the measurement tool itself. Consequently, we emphasize how emotional eating behaviors influence dietary patterns and emotional states at psychological and behavioral levels.

We fully agree with Bongers and Jansen’s perspective that emotional eating scores may not fully capture actual food intake but instead reflect broader dietary behaviors and psychological traits. However, this does not imply that the concept of emotional eating lacks research value. On the contrary, as a psychological and behavioral phenomenon, emotional eating may indirectly contribute to depression risk by shaping individuals’ dietary patterns and emotional regulation capacities. In our findings, emotional eating scores demonstrated significant correlations with body dissatisfaction and depressive symptoms, suggesting a potential mediating role of emotional eating at the psychological level. Although these correlations may not equate to direct causal links with food intake, they remain valuable indicators for assessing individuals’ emotional regulation abilities and eating-related behaviors.

Based on your suggestion, I have deleted the following parts of the manuscript: One explanation is the failure to distinguish between physiological hunger and negative emotions [35]. Another explanation is the cyclic pattern of food-mediated emotion regulation leading to eating [36]; negative emotions are temporarily reduced but then quickly rebounds [37].

In addition, I have added the following to the second and fourth paragraphs of the manuscript's discussion: Our findings highlight the potential psychological role of emotional eating behavior, particularly in regulating emotions and influencing depression risk. Moreover, the results indicate that emotional eating partially mediates the relationship between physical activity and depression. In this context, emotional eating scores may more accurately reflect failures in emotion regulation rather than actual changes in food intake[35]. Consequently, emotional eating is unlikely to result in actual changes in BMI.

Specifically, higher levels of body dissatisfaction are associated with an increased likelihood of engaging in emotional eating behaviors. This finding suggests that body dissatisfaction may indirectly promote emotional eating by reducing self-esteem and self-efficacy, thereby diminishing the motivation to engage in physical activity. In this study, body dissatisfaction specifically refers to dissatisfaction with one's own body weight. When individuals are dissatisfied with their bodies, they may resort to emotional eating as a means of alleviating negative emotions, rather than opting for positive physical activities. This alternative behavior may stem from the immediate gratification provided by emotional eating, as well as the potential physical discomfort or psychological pressure associated with engaging in exercise [42, 43].

**Point 5**

The authors discuss how physical activity and emotional eating partially mediate the risk of depression, but there is not much reflection in the discussion about the explanatory power of each variable or the magnitude of associations observed in the study. The manuscript would be strengthened by translating these numbers into information more easily understood by those who are not experts in structural equation modeling in both the results and discussion sections.

**Response 5**

Thank you very much for your insightful comments regarding the discussion of the explanatory power and the magnitude of associations in our study. We appreciate your suggestion to make the results more accessible to a broader audience by translating the numbers into more easily understood information.

In the first paragraph of the Discussion section, we have added the following content: This study investigates the complex relationships between exercise, body weight dissatisfaction, emotional eating, and depression among Chinese college students using structural equation modeling. Our results demonstrate that exercise significantly mediates the relationship between BMI and depression, accounting for 30% of the total effect. Additionally, exercise mediates the relationship between body weight dissatisfaction and emotional eating, contributing to 19.8% of the total effect. Emotional eating behavior also partially mediates the relationship between exercise and depression.

In the second paragraph of the Discussion section, after the sentence “We found that emotional eating partially mediated the relationship between physical activity and depression (Fig 1).”, we have added the following content: Emotional eating behavior partially mediated the relationship between exercise and depression, accounting for 8.5% of the total effect.

**Point 6**

The authors report data for muscle mass in kilograms in table 1 and report in the methods that muscle mass was assessed by questionnaire. It’s unclear why this measurement, which is arguably more difficult to know and self-report with accuracy than body weight, was not measured whereas body weight and height were measured by the authors. There are accessible tools available for measuring body composition with reasonable accuracy. For example, the Omron HBF-516 home body composition scale has good accuracy when validated against criterion methods (Siedler et al, 2023) and is commercially available and inexpensive. Self-reported muscle mass is unreliable and doesn’t add helpful information to the study.

**Response 6**

In response to the reviewer's comment regarding the measurement of height and weight, we would like to clarify that in the "Measurements" section of our manuscript, we have already described the instruments used for measuring height and weight. Specifically, height and weight were measured by our research staff and reported to the participants, who then recorded these measurements on the questionnaires.

**Minor issues:**

**Point 1**

Abstract: Includes grammatical errors and incomplete sentences, requires review

**Response 1**

Thank you very much for your comments on the abstract section. I have revised the grammatical errors and made the sentences complete.

**Point 2**

At the end of the first paragraph, the authors state, “However, there are few studies on the combined effects of exercise and dietary behaviors on depression.” The authors should support this claim by including citations for these studies.

**Response 2**

Introduction:

Thank you very much for your insightful comments on the concluding sentence of the first paragraph of our manuscript. You pointed out that we failed to provide supporting references when mentioning that there are few studies on the combined effects of exercise and dietary behaviors on depression. You are absolutely right, and this is indeed a crucial point.

In the revised version of our manuscript, we have now added relevant references to support this statement. Here is the newly added content in the text:

For example, a recent systematic review highlighted that while individual studies have examined the links between diet and depression or exercise and depression, the combined effects of these lifestyle factors are less understood [9]. Another study emphasized the need for more research on the integrated impact of physical activity and diet on mental health outcomes [10].

**Point 3**

Paragraph 2, near middle: The authors write, “Research indicates that exercise significantly impacts the severity and relief of depression [14, 15].” Citation 15 appears to be irrelevant to this claim, as the topic of the paper appears to be about immunity.

**Response 3**

Thank you very much for your attention to Citation 15 in the middle of Paragraph 2. You pointed out that Citation 15 appears irrelevant to the claim that “exercise significantly impacts the severity and relief of depression,” as the paper seems to focus on immunity. Your observation is very important, and we appreciate your feedback.

Upon re-evaluation, we realized that the inclusion of Citation 15 was indeed inappropriate. Although the paper touches on the broad health benefits of exercise, its primary focus is not directly related to the relief of depressive symptoms. Therefore, using it as evidence for the impact of exercise on depression is not suitable.

In the revised manuscript, we have removed Citation 15 and added a more relevant reference to support our statement. Here is the revised text:

Research indicates that exercise significantly impacts the severity and relief of depression [14, 15]. For example, a meta-analysis demonstrated that exercise has a moderate antidepressant effect, reducing symptoms of depression across various populations [15]. Similarly, a recent systematic review highlighted a negative dose-response relationship between physical activity levels and depression risk, further supporting the role of exercise in depression management [14].

**Point 4**

Paragraph 3: The authors write in sentence two, “In 2022, according to a study in eating disorders has risen by 80% over the past 5 years [18].” This sentence is incomplete and appears to be missing a word.

**Response 4**

Thank you for pointing out the issue in the second sentence of Paragraph 3. You are correct that the sentence is incomplete and appears to be missing a word. This was an oversight in our manuscript preparation, and we appreciate your attention to detail.

The sentence should read:
“In 2022, according to a study, eating disorders have risen by 80% over the past 5 years [18].”

We have now corrected this sentence in the revised version of our manuscript to ensure clarity and completeness. We apologize for any confusion this error may have caused.

**Point 5**

Paragraph 4: The authors write, “College students body dissatisfaction is closely related to eating behaviors and exercise [24, 25].” Does reference 25 demonstrate this point? Also, very minor, but in this context the word students is possessive and should end with an apostrophe (students’).

**Response 5**

Thank you very much for your insightful comments on Paragraph 4. We appreciate your attention to both the content and the grammatical details. You have raised a valid question about whether Reference 25 effectively supports the statement that “College students' body dissatisfaction is closely related to eating behaviors and exercise.” Upon re-evaluation, we realize that Reference 25, while relevant to body dissatisfaction, does not specifically address the connection between body dissatisfaction and eating behaviors or exercise in college students. We apologize for this oversight.

To address this, we have carefully reviewed the literature and identified a more appropriate reference that directly supports this claim. We have replaced Reference 25 with a study that specifically examines the relationship between body dissatisfaction, eating behaviors, and exercise among college students. Here is the new reference:

Relationship between body dissatisfaction, insufficient physical activity, and disordered eating behaviors among university students in southern China. *BMC Public Health* **2022**, *22* (1), 2054. DOI: 10.1186/s12889-022-14515-9

**Methods**

**Point 6**

Sentence 3: “In accordance with these policies, investigators provide research participants…” Provide should be provided.

**Response 6**

Thank you for pointing out the grammatical error in Sentence 3. You are absolutely right; the verb "provide" should be in the past tense "provided" to maintain consistency and accuracy in the sentence.

We have now corrected the sentence to read:

In accordance with these policies, investigators provided research participants with detailed explanations about the data being collected in this study and confirmed that it is being used only for scientific research purposes and not being disclosed to the public. All participants signed informed consent forms.

**Point 7**

Paragraph 2: What’s head portal sensitization?

**Response 7**

Thank you very much for your comments on the careful observation of the method section. I'm sorry that this is caused by my unclear expression. I have revised the manuscript as follows: Recruitment of participants was conducted through oral publicity and distribution of pamphlets in study rooms.

**Point 8**

Paragraph 3, last sentence: the word “date” should be “data”

**Response 8**

Thank you for your keen observation regarding the last sentence of Paragraph 3. You are correct that the word "date" should be "data." We appreciate your attention to detail.

**Point 9**

C-DEBQ: What’s the range of scores possible on this instrument and are there criterion thresholds for determining what’s a “high” score vs a “low” score? It’s hard to interpret the scores presented in table 1 without context about the range of scores possible.

**Response 9**

Thank you for your insightful question regarding the C-DEBQ (Chinese version of the Dutch Eating Behavior Questionnaire) and the interpretation of the scores presented in Table 1. You are correct that providing context on the range of scores and criteria for determining “high” versus “low” scores is essential for interpreting the results.

I have revised the manuscript as follows: The total score for each dimension is calculated by summing the scores of the respective items. The possible range of scores for each dimension is as follows: emotional eating (13-65), external eating (10-50), and restrained eating (10-50). Higher scores indicate a greater degree of the specific eating behavior.

**Point 10**

Directly after the C-DEBQ description: The sentence stating “A questionnaire was utilized….monthly living expenses, and other relevant details” should be moved to the start of the methods with the other general information assessed by questionnaire. Also, the phrase “and other relevant details” is vague and not repeatable. The authors should include specific information for all details assessed on the questionnaire, or provide justification for why the specific nature of the additional information is not listed.

**Response 10**

Thank you for your detailed feedback regarding the placement and clarity of the sentence describing the questionnaire used in our study. We appreciate your suggestions for improving the structure and specificity of our methods section.

We agree that the sentence describing the questionnaire should be moved to the beginning of the Methods section, alongside other general information assessed by questionnaire. This will improve the flow and organization of the section.

We acknowledge that the phrase “and other relevant details” is vague and not conducive to reproducibility. To address this, we have revised the sentence to include specific information about all details assessed in the questionnaire. This ensures that our methods are transparent and repeatable.

I have revised the manuscript as follows:

The questionnaire collected students' basic information, including age, muscle mass, gender, height, weight, body dissatisfaction index and monthly living expenses. Additionally, it comprised three scales to assess exercise levels, one to measure emotional states, and one to evaluate eating behaviors.

The factor of average monthly living expenses was not included in the final statistical analysis and is not reflected in Table 1.

**Point 11**

Section 2.5, Data Collection: The authors note that they removed invalid questionnaires. What’s the basis for determination of invalidity?

**Response 11**

Thank you for your question regarding the exclusion criteria in Section 2.1. We appreciate the opportunity to clarify this point.

In the third paragraph of Section 2.1, we established the exclusion criteria. Given that our study focuses specifically on college students, individuals over 24 years of age were excluded. This decision was made because those above 24 years old are more likely to be postgraduate students rather than undergraduates. Our study aims to investigate the specific population of undergraduate students, and thus, we chose to exclude this older age group to maintain the homogeneity of our study sample.

**Results**

**Point 12**

Paragraph 1, sentence 2 begins “Man” and should read “Men.” This paragraph ends with information about the mean SDS scores being higher in women than men. However, it’s notable that, according to the data in table 1, a slightly greater proportion of males (74.1%) were classified as depressed than were females (69.4%) and this difference was statistically significant (p=0.032) but this was not noted in the text of the results.

**Response 12**

Thank you very much for pointing out the error and for highlighting the discrepancy between the text and the data presented in Table 1. We appreciate your attention to detail and acknowledge the importance of accurately reporting our findings.

We have added the following after the first paragraph of the results section:

Notably, a slightly greater proportion of males (74.1%) were classified as depressed compared to females (69.4%), and this difference was statistically significant (p=0.032). This finding suggests that while women had higher SDS scores overall, the prevalence of depression was slightly higher in men.

**Point 13**

There appear to be typos in table 1 where data are presented as n (%). The numbers presented do not equal the percentage of the total sample indicated in the parentheses for values in the data for BMI category, activity level category, and SDS category.

**Response 13**

Thank you very much for your meticulous observation. I am not sure why a digit was missing from the numbers in the table after I transferred it into the manuscript. I sincerely apologize for this oversight.

I have now revised Table 1 to ensure that all data are accurate and complete.

**Point 14**

Table 2 does not report information about measurement error.

**Response 14**

Thank you very much for your comments on Table 2. I have read the paper by Kang and Ahn (2021) and noticed that Table 2 in their paper is similar to Table 2 in my article. However, the variables x1, x2, x3, etc., in their paper are observed variables, while the variables in my paper are latent variables. Additionally, Table 2 in my paper uses Pearson correlation analysis, not structural equation modeling. If you need to view the standard deviations (SD) of these variables, they are provided in Table 1.

**Point 15**

As described earlier in the major issues section, the results section would be stronger if it included data tables with information about the model fit and measurement model, such as that presented in Kang and Ahn, 2021, tables 3 and 4.

**Response 15**

Thank you very much for your comments on the methods section of the manuscript. Based on your comments, I found the paper you mentioned and reported the relevant fit index. The following additions were added to the manuscript: Moreover, the model fitting index indicated that the structural equation model was satisfactory (AIC = 56057, BIC = 56155, P = 0.013). Table 4 provides examples of reporting results that mainly use fit indices. We used several fit indices to test the fit of the data model: (i) Fit index (CFI, AGFI, GFI, and TLI) >0.95 was considered satisfactory, and values between 0.90 and 0.95 were considered acceptable; (ii) Root mean square Error of Approximation (RMSEA), the range of 0.05-0.08 indicates a sufficient fit; Standardized root mean square residual (SRMR), values < 0.05 indicate a good fit and a range between 0.05 and 0.10 is acceptable.

Table 4. Goodness of Fit Indices

|  | χ² | df | GFI | AGFI | RMSEA | SRMR | CFI | TLI | |
| --- | --- | --- | --- | --- | --- | --- | --- | --- | --- |
| Model | 8.632 | 2 | 0.998 | 0.985 | 0.043 | 0.009 | 0.996 | 0.982 |  |

Note. AGFI = adjusted goodness of fit index; CFI = comparative normed fit index; df = degree of freedom; GFI = goodness-of-fit index; RMSEA = root mean squared error of approximation; SRMR = standardized root mean square residual; TLI = Tucker-Lewis index.

**Discussion**

**Point 16**

The manuscript would be strengthened by a sentence or two at the start of the discussion summarizing the key findings of this paper as they relate to the specific aim and hypothesis of this study and what it adds to the literature before going into deeper discussion of the study.

**Response 16**

Thank you very much for your comments on the discussion section. I have added the corresponding content in the first paragraph of the discussion. As follows: This study investigates the complex relationships between exercise, body dissatisfaction, emotional eating, and depression among Chinese college students using structural equation modeling. Our results demonstrate that exercise significantly mediates the relationship between BMI and depression, accounting for 30% of the total effect. Additionally, exercise mediates the relationship between body dissatisfaction and emotional eating, contributing to 19.8% of the total effect. Emotional eating behavior also partially mediates the relationship between exercise and depression.

**Point 17**

In paragraph 2, the authors discuss how exercise plays a role in the model. The second sentence notes the “potential of positive exercise in mitigating depression risk.” It’s unclear what the authors mean by “positive” exercise. Please clarify or reword. Additionally, in sentences 4 and 5 of this paragraph the authors state that their results contradict prior studies indicating that exercise reduces BMI. However, the authors conducted cross sectional study of participants, no conclusions can be drawn about the temporality of the relationship between BMI and exercise since reverse causality is possible, i.e. higher BMI individuals recently started exercising more frequently in effort to change body weight. It seems that this may be what the authors are suggesting in sentence 7, but it could be reworded for clarity.

**Response 17**

Thank you very much for your detailed feedback and suggestions regarding Paragraph 2. We appreciate your insights and will address each point to clarify and improve the text.

Revised Paragraph 2:

Our model not only identified the mediating effect of emotional eating but also found that exercise plays an important role in the model. We found that physical exercise has the potential to mitigate depression risk (Table 2). A UK analysis of 15 prospective studies—including a systematic review involving nearly 200,000 adults—corroborated our results, revealing a negative dose-response relationship between physical activity and depression, with a steeper gradient as physical activity levels decreased [14]. Our model suggests that exercise's salutary impact on depression partially stems from its association with BMI, with higher-BMI individuals more inclined toward exercise. Notably, this contradicts prior studies indicating exercise reduces BMI [36]. In recent years, economic downturn, internet usage, and high-pressure educational methods have all contributed to people not having enough time for physical activities [37-39]. Coincidentally, the overall level of physical activity among our participants was considerably low, but some participants who were highly obese strengthened their physical activity, leading to the occurrence of this result. However, it is important to note that our study is cross-sectional, and we just draw conclusions about the temporality of the relationship between BMI and exercise. Reverse causality is possible, meaning that individuals with higher BMI might have recently increased their physical activity levels in an effort to change their body weight. Further longitudinal studies are therefore needed to establish the direction of causality.

**Point 18**

Paragraph 3, sentence 3: This sentence is confusing and appears to be missing some words and/or punctuation, needs revised for clarity.

**Response 18**

Thank you for your feedback regarding Sentence 3 in Paragraph 3. We acknowledge that the sentence is confusing and requires revision for clarity. We appreciate your attention to this issue and have revised the sentence accordingly.

I have amended this in the discussion section of the manuscript: Similarly, a study of over 1,300 Southern Chinese college students revealed that 52.7% reported high levels of emotional eating, and the prevalence of depressive symptoms was 18.6%. The study also demonstrated a strong correlation between emotional eating and depressive symptoms [43], which aligns with our findings.

**Point 19**

In paragraph 4, the authors argue that the transition to college causes changes in eating behaviors and that women and men cope differently and cite three studies for support (#44, 45, 46). This makes me wonder who are the populations studied in these papers, and are students in China expected to respond in similar ways, or could cultural differences result in other coping behaviors that may be different? Sociocultural factors have strong influences on eating behaviors and practices.

**Response 19**

Thank you for your insightful comments regarding Paragraph 4. We appreciate your attention to the potential influence of sociocultural factors and the need to consider cultural differences in coping behaviors.

The sentence, “The transition to college life can be stressful for many women, leading them to resort to eating behaviors to relieve stress [40],” is intended to support the finding that women scored higher on emotional eating than men (Table 1). This statement aims to provide a potential explanation for the observed gender differences in emotional eating behaviors.

Thank you for your suggestion. I agree that incorporating the influence of sociocultural factors can strengthen the argument. Here is the revised paragraph with the added content: Moreover, Society holds higher expectations and stricter norms for women's body image and eating behaviors. Women are more frequently exposed to negative information about weight and appearance, which may lead to higher levels of emotional eating behaviors [48].

After the revision, this paragraph argues that women scored higher on emotional eating than men from three perspectives: the practical reality, the psychological differences between men and women, and the sociocultural differences affecting men and women.

**Point 20**

Paragraph 5: The authors write near the middle of the paragraph, “Perhaps a relatively large proportion of our survey respondents experienced body dissatisfaction that then triggered a qualitative change in the results. Second, unlike most other research methods, the current research methods employed in the actual and ideal BMI difference for representing body dissatisfaction may also lead to identifying different causes.” It’s unclear what the authors mean by triggered a qualitative change in the results. Additionally, related to earlier concerns about the validity of the body dissatisfaction metric used in this study, there is no justification in the methods for why the authors chose their metric for body dissatisfaction instead of a method used in prior studies on body dissatisfaction, and the discussion of this potential limitation is restricted to the two sentences quoted above and the subsequent two sentences in the discussion. Elaboration would strengthen the manuscript.

**Response 20**

Thank you for your detailed feedback and suggestions regarding Paragraph 5. Upon careful consideration, we have found that this paragraph is repetitive of our earlier discussions. Therefore, we have decided to remove this entire section to avoid redundancy and improve the clarity and conciseness of our manuscript.

**Point 21**

Limitations of the study: Elaboration in this section, or integrating an expanded discussion of limitations within the other text of the discussion, would strengthen the manuscript. Also, there is a typo in sentence 3; the word mouth should be month.

**Response 21**

Thank you very much for your constructive feedback regarding the elaboration of the limitations section and the typo in Sentence 3. We appreciate your attention to detail and agree that a more comprehensive discussion of the limitations will strengthen our manuscript.

Revised Limitations Section:

This study had several limitations. With 1,714 participants, the results may lack generalizability. Moreover, only Chinese college students were included, which restricts the applicability of these findings to other countries or age groups. Exercise intensity in the past month was subjectively determined by participation via a questionnaire [53], making it challenging to quantify the subsequent effect of these activities on depression. Additionally, our study is unable to control for confounding variables, such as socioeconomic status, nutrition, and environmental exposures. Therefore, our study has certain limitations in drawing conclusions. Our study employed a cross-sectional design, which limits our ability to draw conclusions about the temporality of the relationships between body weight dissatisfaction, exercise, emotional eating, and depression. Future longitudinal studies are needed to establish the direction of causality and to better understand the dynamic interplay among these variables over time. Body weight dissatisfaction was measured using the difference between actual and ideal BMI. While this method provides a straightforward and quantifiable measure, it may not capture the full complexity of body dissatisfaction as assessed by more detailed psychological instruments. Future studies may consider using additional measures, such as self-report questionnaires or qualitative assessments, to provide a more comprehensive understanding of body dissatisfaction. While our findings suggest that addressing body weight dissatisfaction may be beneficial for improving mental health, our study did not test specific interventions. Future research should explore targeted interventions to reduce body dissatisfaction and emotional eating, and to promote physical activity among adolescents and young adults.
